# Supplementary material for: Micropillar arrays, wide window acquisition and AI-based data analysis improve comprehensiveness in multiple proteomic applications
Source: Nat Commun. 2024 Feb 3;15:1019. doi: 10.1038/s41467-024-45391-z (PMC10838342; doi:10.1038/s41467-024-45391-z)
Supplement: Supplementary file 1 — Supplementary Information [file 41467_2024_45391_MOESM1_ESM.pdf]

Micropillar arrays, wide window acquisition and AI-based data analysis improve comprehensiveness in multiple proteomic applications

Manuel Matzinger[1,*,§], Anna Schmücker[2,3,4], Ramesh Yelagandula[2,5,6], Karel Stejskal[1,2,5], Gabriela Krššáková[1,2,5], Frédéric Berger[2], Karl Mechtler[1,2,5,§], Rupert L. Mayer[1,*,§]

[1] Research Institute of Molecular Pathology (IMP), Vienna BioCenter, Vienna, Austria.
[2] Gregor Mendel Institute of Molecular Plant Biology (GMI), Austrian Academy of Sciences, Vienna BioCenter (VBC), Vienna, Austria.
[3] MRC (Medical Research Council) London Institute of Medical Sciences, Du Cane Road, London, W12 0NN, UK
[4] Institute of Clinical Sciences, Imperial College London, Hammersmith Hospital Campus, Du Cane Road, London, W12 0NN, UK
[5] Institute of Molecular Biotechnology (IMBA), Austrian Academy of Sciences, Vienna BioCenter (VBC), Vienna, Austria.
[6] Laboratory of Epigenetics, Cell Fate & Disease, Centre for DNA Fingerprinting and Diagnostics (CDFD), Uppal, Hyderabad, India.

* These authors contributed equally

§Correspondence to: manuel.matzinger@imp.ac.at, karl.mechtler@imp.ac.at and rupert.mayer@imp.ac.at

KEYWORDS: single cell proteomics, mass spectrometry, liquid chromatography, affinity purification, chromatin remodelers, Smarca5, Arid1a

Supplementary Information

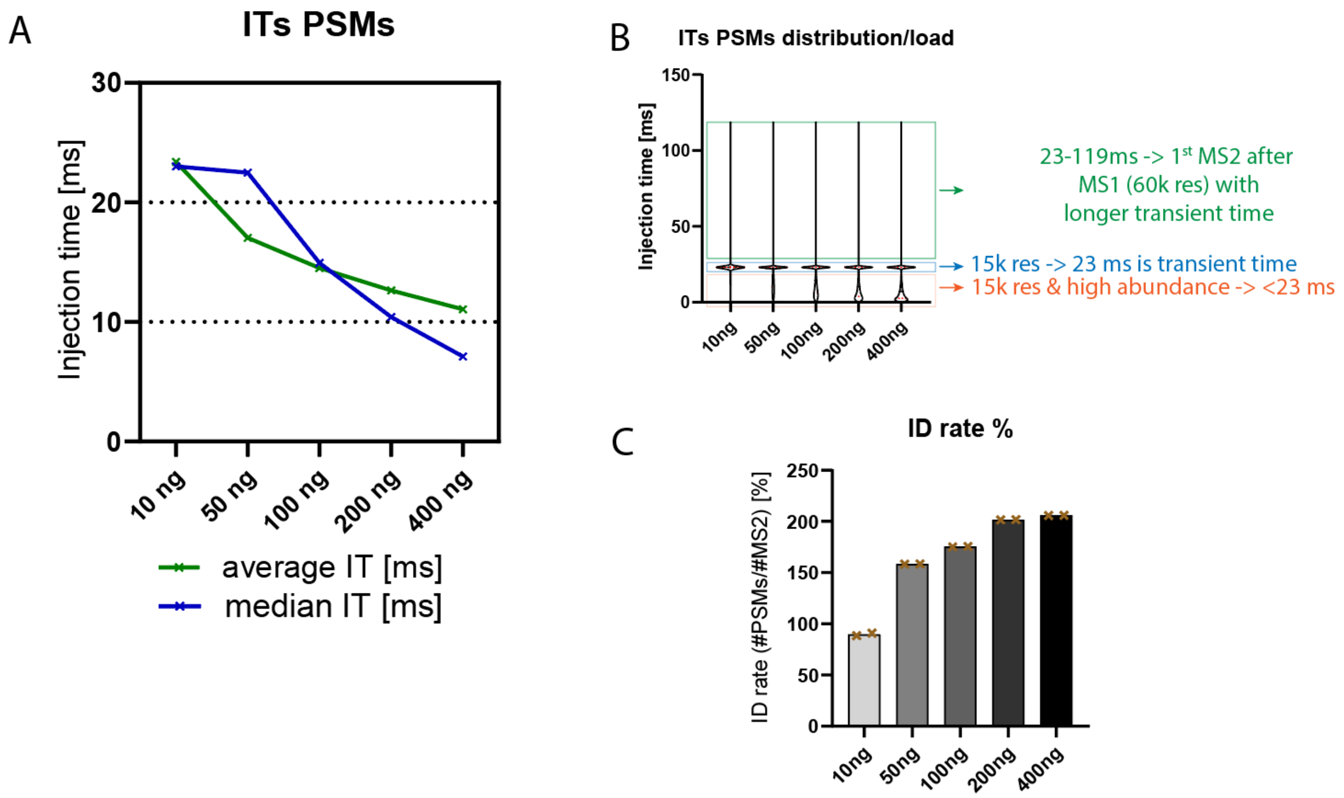

Supplemental Figure 1: ***Injection time and identification rate survey for different input amounts***. *MS2 injection times (ITs) and identification (ID) rates for the 30min gradient with the 50 cm neo column were plotted for peptide spectrum matches (PSMs) for better estimation of ideal maximum MS2 injection time.* ***(A)*** *Shows the respective average (green) and median (blue) MS2 injection times and indicates that for 10ng of the triple proteome mix (HeLa:yeast:E. coli) the maximum fill times of 23 ms are reached and longer maximum fill times would have resulted in more identified peptides. The violin blot in* ***(B)*** *indicates the distribution of the injection times with higher fill times than 23ms only being reached for MS2 spectra directly following higher resolution (60k) MS1 scans allowing a maximum fill time of 119 ms. With increasing injection amount the distribution shifts as expected to lower fill times.* ***(C)*** *illustrates the identification rate to indicate success rate per MS2 spectrum. While the ID rate remains essentially unchanged from 200 to 400 ng, again the low ID rate of 80% for 10ng could potentially be pushed higher when using longer MS2 fill times and potentially also wider isolation windows than m/z 4.*

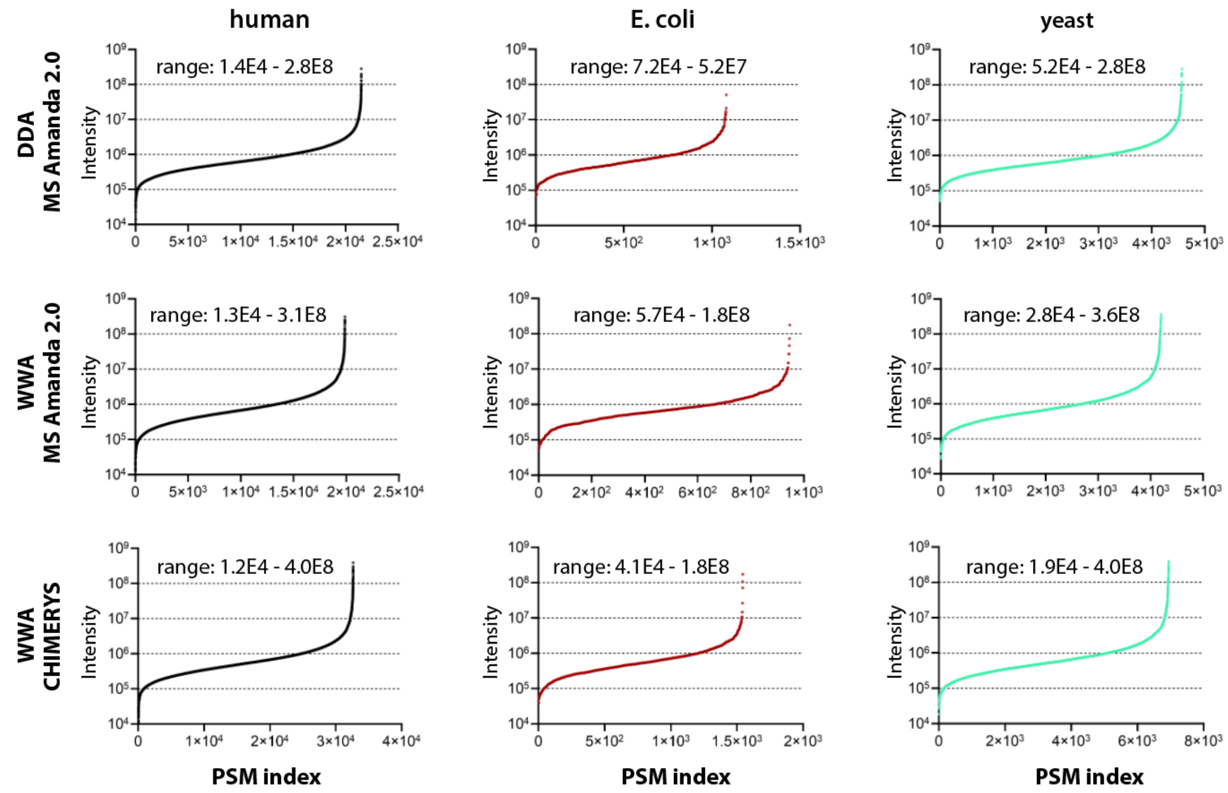

Supplemental Figure 2: ***Data analysis using CHIMERYS improves the dynamic range of identifiable tryptic peptides****. The identifiable peptide pools in the triple proteome mix samples between classical DDA acquisition using MS Amanda 2.0 and WWA using CHIMERYS were compared by ranking PSMs from lowest to highest abundant one and assigning a corresponding PSM index, indicating that the later approach allows the identification of peptides of a greater dynamic range in general when compared to the classical approach. n = number of quantified PSMs per condition, n = 21,497 for human DDA Amanda, n = 19,870 for human WWA Amanda, n = 32,570 for human WWA CHIMERYS, n = 1,081 for E. coli DDA Amanda, n = 947 for E. coli WWA Amanda, n = 1,542 for E. coli WWA CHIMERYS, n = 4,570 for yeast DDA Amanda, n = 4,199 for yeast WWA Amanda and n = 6,934 for yeast WWA CHIMERYS.*

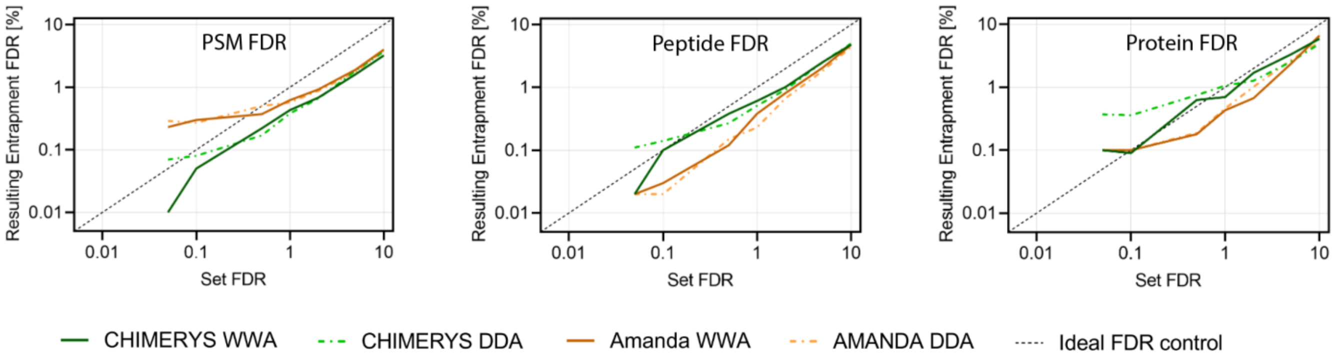

Supplemental Figure 3: ***Entrapment experiments confirm reliable FDR control of CHIMERYS and MS Amanda 2.0****. Two mouse co-immunoprecipitation samples were recorded with a precursor isolation width of 1 (DDA) and 4 (WWA). Raw files were subsequently searched with a target database and an additional custom-made decoy database to estimate the FDR control of CHIMERYS and MS Amanda 2.0. The results for all runs and both software packages demonstrate excellent FDR control on all three levels, PSM, peptide and protein level from 1% upwards. Moreover, all analyses, except the CHIMERYS results for the DDA run, display excellent FDR control already from 0.1% upwards on peptide and protein level. n = 1 per condition.*

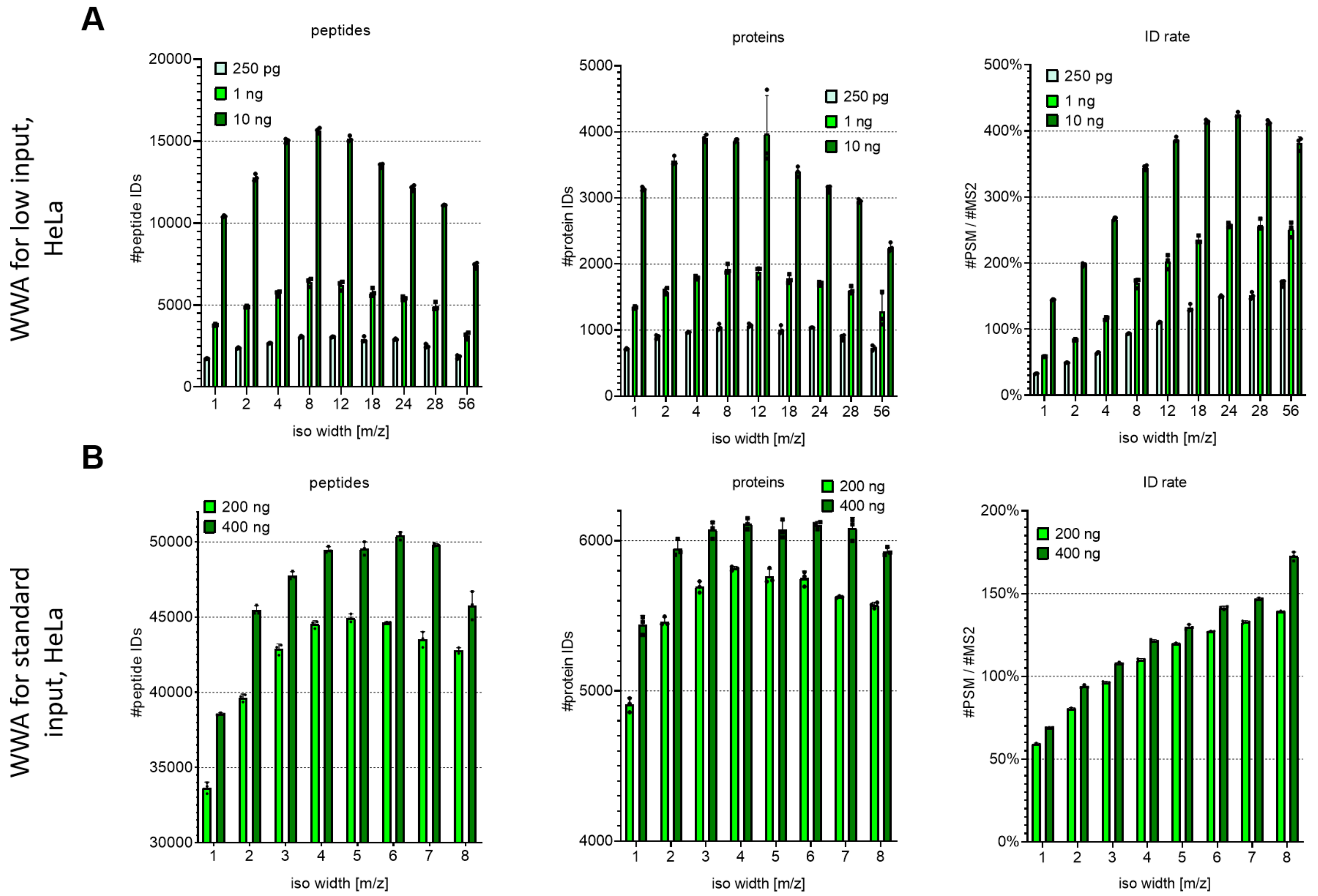

Supplemental Figure 4: ***Ideal precursor window size for wide window acquisition for optimal utilization of CHIMERYS depends on injected sample amount.*** *Different isolation window sizes were tested to identify the most well-suited precursor isolation window size for* ***(A)*** *low sample input from 250 pg up to 10 ng measured on the 5.5 cm column, and* ***(B)*** *standard sample inputs of 200 and 400 ng measured on the 50 cm column. Results are shown for peptide and protein IDs as well as identification rate (ID rate). ID rates were calculated by dividing the number of obtained peptide-spectrum matches (PSM) by the number of recorded MS2 spectra. ID rates over 100% indicate that more than one peptide could could be identified per (chimeric) spectra on average. Bars indicate means and error bars indicate standard deviations, n= 3 technical replicates.*

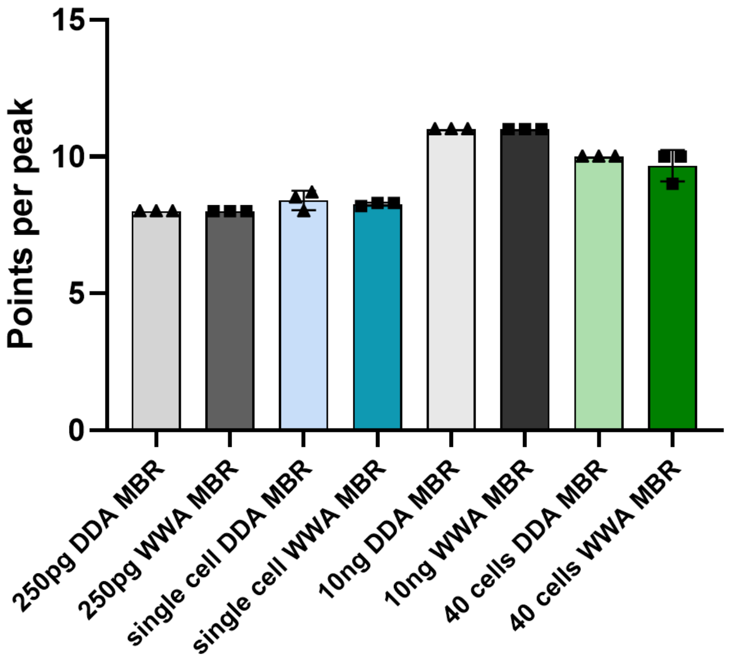

Supplemental Figure 5: ***DDA and WWA show consistent number of datapoints over the peak for low input samples.*** *Low input HeLa bulk runs and single cell runs were assessed for the respective median points per peak as provided by apQuant and literally no differences between DDA and WWA could be quantified when matching-between-runs was enabled and files were searched together per sample type (DDA 250 pg + 10 ng, DDA single cells + 40 cells, WWA 250 pg + 10 ng and WWA single cells + 40 cells). n = 3 replicates/condition.*

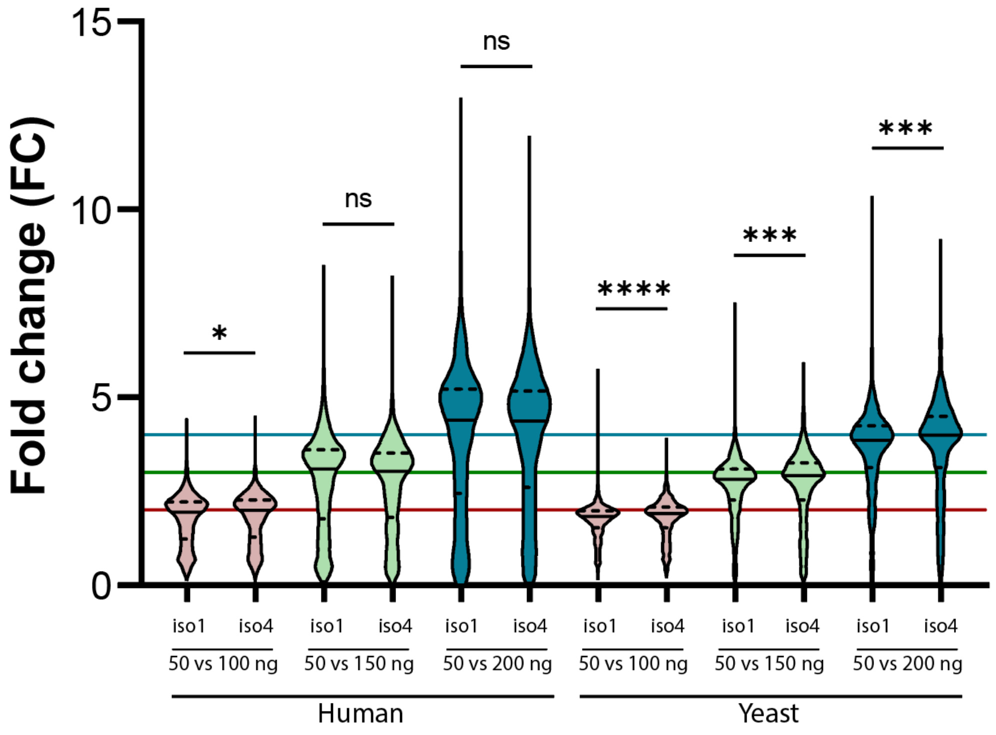

Supplemental Figure 6: ***Both, DDA and WWA allow accurate relative protein quantification for regular input samples.*** *Double proteome samples of commercial HeLa and yeast digests with a constant total peptide amount of 200 ng were prepared with varying relative compositions and analyzed with DDA (isolation width 1) and WWA (isolation width 4) and fold changes per protein between different abundant samples calculated. Colored horizontal lines represent the respective expected fold changes. Comparing medians of measured fold changes against expected fold changes showed small deviations from in the range of 0.2-9.8 %. WWA resulted in slightly improved quantitative accuracy for all comparisons, while this effect was more evident for yeast proteins (see also Supplemental File 2). No data set passed Shapiro-Wilk normality testing, hence unpaired, two-sided Mann-Whitney testing was performed for assessment of differences between means. n = Number of protein fold changes per condition in order of appearance from left to right 1,370, 1,385, 1,398, 985, 999, 1,007, 1,341, 1,352, 1,355, 910, 915, and 908.* ***(A-D)*** ** p value ≤0.05 *** p value ≤0.001 **** p value ≤0.0001 ns p value >0.05. Source data are provided as a Source Data file. Exact p values from left to right: 0.0341, 0.0872, 0.9362, <0.0001, 0.0010, 0.0004.*

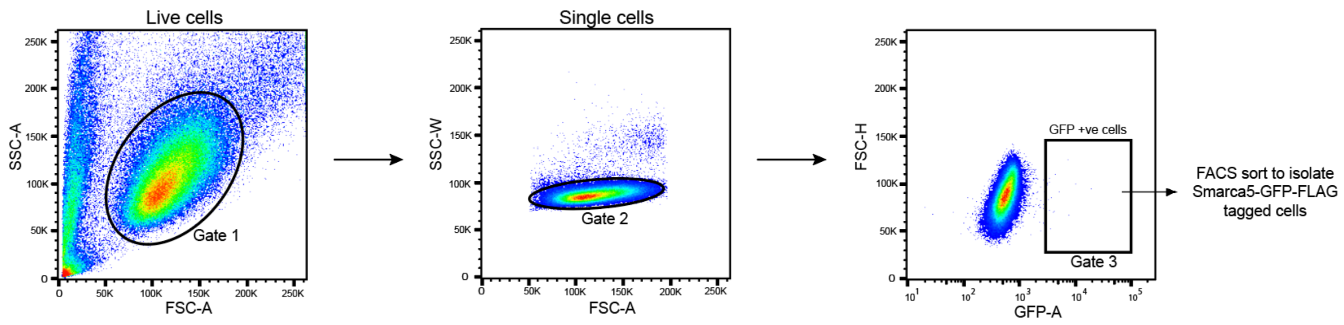

Supplemental Figure 7: ***Gating strategy for isolation of Smarca5-GFP-FLAG expressing mouse ES cells.*** *For generation of the FLAG tagged Smarca5 cell line, transfected mouse embryonic stem cells were recovered for two days and sorted via FACS. Live cells were first selected according to cell granularity (via side scatter SSC-A) and cell size (via forward scatter FSC-A). Single cells were then further selected by gating via side scatter pulse width (SSC-W) and FSC-A. Finally, only strongly GFP-expressing single cells were isolated by gating via forward scatter pulse height (FSC-H) and GFP-A channel intensity.*
