# Supplementary material for: Micropillar arrays, wide window acquisition and AI-based data analysis improve comprehensiveness in multiple proteomic applications
Source: Nat Commun. 2024 Feb 3;15:1019. doi: 10.1038/s41467-024-45391-z (PMC10838342; doi:10.1038/s41467-024-45391-z)
Supplement: Supplementary file 3 — Description of Additional Supplementary Files [file 41467_2024_45391_MOESM3_ESM.pdf]

Description of Additional Supplementary Files

File Name: Supplementary Data 1

Description: **Description of employed LC gradients for the µPAC column comparisons.** Column A depicts the column lengths for which the respective gradients were employed. Column B shows the column volumes, while column C gives the gradient length and column D the injection mode, which was direct injection without the use of a trap column for all analyses in the course of the µPAC column comparisons. Column E depicts the loading setting which was either combined control restricintg the flow rate and the pressure or pressure control only. The injection volume presented in F was in all cases around 6 µL (0.5-1 µL sample + additional 5 µL of loading solvent). Average times for sample aspiration and loading are given in G as flow and/or pressure control thresholds lead to slight differences in loading times and consequently overall run times. Columns H-N describe the increase in organic solvent over time during the gradient starting at 99% buffer A (0.1% formic acid) and 1% buffer B (80% acetonitrile, 0.08% formic acid). Column J describes the time at which 22.5% buffer B was reached, while column L indicates the gradient time at which 40% solvent B was reached. Flowrates used throughout the gradient are given in column N. The time at which 95% buffer B concentration was reached for column washing is detailed in column O, and the time until the washing was carried out is presented in column Q. Washing flowrates are given in column S with increasing flowrates for the shortest methods. Details on column equilibration are given in columns T-X. The overhead times (without washes) are presented in column Y and the total run-to-run times in column Z.

File Name: Supplementary Data 2

Description: **Assessment of quantitative reproducibility of HeLa low input and single cell data.** For the assessment of quantitative reproducibility for low input and single cell HeLa data, coefficients of variation (CVs) were calculated for the apQuant areas per sample condition for i) analyses per individual raw file, and ii) analyses with matching to 10 ng for the 250pg bulk samples or the 40 cells for the single cells samples. CVs per protein were calculated for the three samples per condition with the highest number of quantified proteins. WWA led to a higher number of proteins that could be quantified in all of the three assessed replicates. WWA also led to lower average CVs and therefore better quantitative reproducibility for 250 pg, single cells and 40 cells. Excel file with 17 tabs with tabs B-Q presenting the quantitative reproducility for each condition.

File Name: Supplementary Data 3

Description: **Assessment of quantitative accuracy using HeLa/yeast double proteome mixes.** For the assessment of quantitative accuracy for regular input samples, 200 ng total input amount consisting of different ratios between HeLa and yeast digest were analyzed with a classical DDA approach using 1 m/z isolation width as well as WWA using 4 m/z isolation width. All samples contained in total 200 ng that were injected on column with the following different ratios (ng HeLa/ng Yeast): 200/0, 150/50, 100/100, 50/150, 0/200. Expected fold changes were compared to calculated fold changes between samples of different HeLa/yeast ratios. This Supplementary Data file consists of 17 tabs. Tab A shows an overview of all quantitative comparisons using both 1 and 4 m/z isolation widths. Deviations from the expected ratios are given in column G as % error between the median measured

ratios and the expected ratios. Tab B and C show overviews of the expected against the experimental fold changes between samples of different ratios for tab B isolation with 1 and tab C isolation width 4. Tabs D and K illustrate all identified proteins for runs with isolation width 1 (tab D) and isolation width 4 (tab K). In tabs E-J protein fold changes were calculated for human (tabs E-G) and yeast (tabs H-J) proteins for isolation width 1. In tabs L-Q protein fold changes were calculated for human (tabs L-N) and yeast (tabs O-Q) proteins for isolation width 4.

File Name: Supplementary Data 4

Description: **Reanalysis of Furlan et al. 2019 raw data using CHIMERYS and apQuant and comparison to originally identified interactors.** AP-MS data from Furlan et al. 2019 was reanalyzed to assess the bioinformatic part of the proposed advanced analytical workflow. Therefore, raw data was downloaded from PRIDE repository with the accession number PXD012800. Raw files for the bait proteins SMC1A and CDK8 were downloaded and reanalyzed. The raw files for two different baits at three different inputs (4ug and 12,000 cells for SMC1A and 25,000 cells for CDK8) were reanalyzed using CHIMERYS and apQuant and compared to the original data provided by Furlan et al. here (https://www.nature.com/articles/s41467-019-09533-y#Sec17). Excel file with 11 tabs listing all proteins found to be interactors at 1% FDR.

File Name: Supplementary Data 5

Description: **Co-immunoprecipitation analysis to identify novel interactors of mouse Smarca5 in mouse embryonic fibroblasts.** Excel file with 5 tabs listing all identified proteins for the classical analysis workflow (tab B) and the advanced analysis workflow (tab C). Proteins with significantly higher abundance in the flag-tagged co-IP samples (5% FDR) are denoted as potential Smarca5 interactors and listed in this table combined (tab A) as well as separately with more details in tabs D and E. Proteins highlighted with red background represent potential interactors quantified using a single peptide only and therefore indicate low abundance or less confident potential interactors. Highlighted in red and bold font is the bait protein Smarca5, which was identifed at higher abundance in flag-tagged co-IP samples using both classical and advanced workflow. Proteins denoted with green background represent known Smarca5 interactors as indicated on https://string-db.org/ in February 2023 (https://version-11-5.string-db.org/), which have additionally been quantified with ≥2 peptides.

File Name: Supplementary Data 6

Description: **Description of MS methods for the µPAC column comparisons.** Details for the mass spectrometry methods employed to compare the different micro pillar array columns are described in this supplemental table. Column A denotes the unique raw file name tag associated with each set of triplicates per condition. Column B, C and D illustrate the injection amount the LC gradient length and the applied chromatographic column. Columns E and F describe the applied solvent flow rate for the LC and the FAIMS compensation voltages in Volt. Columns G and H give the precursor isolation width and duty cycle duration/ FAIMS compensation voltage. Columns I, J and K report the MS1 resolution, the MS1 injection time and the MS1 target, which were held constant for all employed methods. The duration for how long a fragmented precursor is to be exluded from further fragmentation is given in column L as well as if precursors should be excluded for all compensation

voltages (=shared). MS2 resolution is reported in column M, which was held constant at 15,000 for all methods. Columns N, O and P present the fragmentation collision energy, MS ion injection time the MS2 target value, which were held constant for all different methods. The overall MS method duration is given on column R.

File Name: Supplementary Data 7

Description: **Full STRING DB analysis of all identifed potential Smarca5 interactors.** All potential Smarca5 interactors identifed from the co-immunoprecipitation experiments were submitted to STRING (https://string-db.org/) in three separate batches: i) only potential interactors unique to the classical workflow, ii) potential interactors shared between workflows, and iii) potential interactors unique to the advanced workflow. Smarca5 was added to each submission as reference point. The network type was set to physical subnetwork and network edges were set to confidence. All other settings were kept at default. As background all identifed proteins for the classical workflow were used for the classical workflow-specific interacotrs, and for the shared and advanced workflow-specific interactors all proteins identifed by the advance platform were used. STRING-DB was accessed on March 1st 2023.
